# 

**Published:** 1890-10-11

**Authors:** 


WITH EXTRA NURSING SUPPLEMENT.

Per Annum, 6s. 6d.; post free.

Monthly Parts, 6d.; post free 8d.

Ditto per Annum, 8s., post free.

### THE HOSPITAL

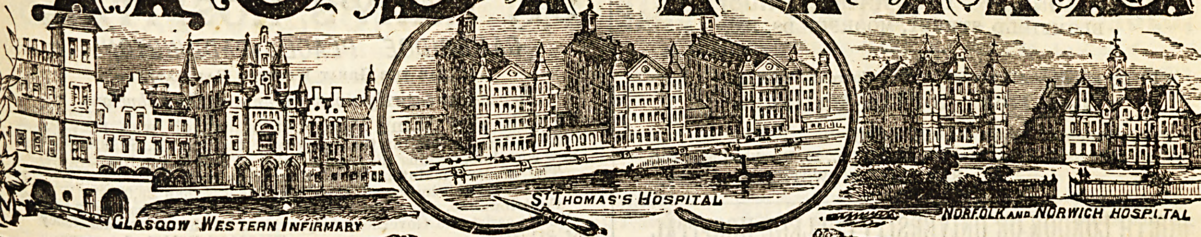

A WEEKLY JOURNAL OF

Science, Medicine, Nursing and Philanthropy.

### SATURDAY, OCTOBER 11th, 1890.

**The Administrative Advance** - 17
**Words of Consolation.**—Thanksgiving - 18
**The Doctor's Pulpit.**—Are Lady Doctors Clever? - 19
**House Ventilation and Warming** - 20
**The Story of the Insane from Year to Year** - 20
**A Celebrated Matron** - 21
**Manual Training in Schools.**—II. In America, Sweden, and France - 23
**Notes and News** - 24
**Annotations.**—Costers to the Rescue—Patients' Dinners—Harvest Festivals and Hospitals—The Winter Session - 26
**The Practitioner's Mirror and Retrospect:—**
Medicine—Starvation in Enteric Fever—Curability of Hepatic Cirrhosis by Iodide of Potassium and Jaborandi—Death from Burns—Saline Subcutaneous Injections - 27
Surgery—Thyroid Grafting—Resection of the Liver - 28
Therapeutics—Starving - 28
**October** - 29
**Working Women in Large Towns.**—XII. Economic Aspects of the Subject - 30
**General Charities** - 31
**Passing Topics.**—Clap-trap Criticism - 32
**Scraps and Gleanings** - 32

### EXTRA SUPPLEMENT—THE NURSING MIRROR.

**En Passant.**—A Letter to Nurses—The Cancer Hospital, Fulham Road—Battle—Short Items—The First Thousand—A Tribute of Praise—London Hospital Nurses—A Trained Nurses' Agency - v
**Sketches of Insanity.** By F. H. Walmsley, M.D.—XIII. Alcoholic Insanity - vi
**Notes from Australia** - vii
**Death in Our Ranks** - vii
**Presentation** - vii
**Everybody's Opinion.**—The Nurses of the London Hospital - viii
**Lectures and Amusements—Notes and Queries** - viii
**Vacancies—Amusements and Relaxation** - viii

